# A comparison of sampling methods for examining the laryngeal microbiome

**DOI:** 10.1371/journal.pone.0174765

**Published:** 2017-03-31

**Authors:** Alissa S. Hanshew, Marie E. Jetté, Stephanie Tadayon, Susan L. Thibeault

**Affiliations:** 1 Department of Surgery, University of Wisconsin School of Medicine and Public Health, Madison, Wisconsin, United States of America; 2 Environmental Health and Safety, The Pennsylvania State University, University Park, Pennsylvania, United States of America; 3 Department of Otolaryngology, University of Colorado School of Medicine, Aurora, Colorado, United States of America; Argonne National Laboratory, UNITED STATES

## Abstract

Shifts in healthy human microbial communities have now been linked to disease in numerous body sites. Noninvasive swabbing remains the sampling technique of choice in most locations; however, it is not well known if this method samples the entire community, or only those members that are easily removed from the surface. We sought to compare the communities found via swabbing and biopsied tissue in true vocal folds, a location that is difficult to sample without causing potential damage and impairment to tissue function. A secondary aim of this study was to determine if swab sampling of the false vocal folds could be used as proxy for true vocal folds. True and false vocal fold mucosal samples (swabbed and biopsied) were collected from six pigs and used for 454 pyrosequencing of the V3–V5 region of the 16S rRNA gene. Most of the alpha and beta measures of diversity were found to be significantly similar between swabbed and biopsied tissue samples. Similarly, the communities found in true and false vocal folds did not differ considerably. These results suggest that samples taken via swabs are sufficient to assess the community, and that samples taken from the false vocal folds may be used as proxies for the true vocal folds. Assessment of these techniques opens an avenue to less traumatic means to explore the role microbes play in the development of diseases of the vocal folds, and perhaps the rest of the respiratory tract.

## Introduction

The role of bacterial communities in human health has been extensively researched in the past few decades, particularly for easily sampled body sites such as skin [1], mouth [2], nose [3], and the intestinal tract[4]. Common sampling methods include mucosal biopsy[5], brush[6], and swab[7], as well as lavage[8] and fecal collection [9]. The vocal folds are mucosal tissues housed within the larynx at the junction between the respiratory and gastrointestinal tracts and there is evidence that they have a distinct immunologic role [10] and microbial community[11]. The primary function of the vocal folds is airway protection; however, their vibrational properties result in voice production, making them an essential tool of human communication. Studying the microbiota by procuring biopsies of vocal fold tissue from live humans is not ethically feasible, as even small tissue deficits have negative effects on voice[12] and it is not currently possible to regenerate vocal fold tissue. Therefore, in the literature addressing the human laryngeal microbiome, researchers have relied on tissue sampling of mucosal biopsies adjacent to the vocal folds[11, 13] or benign vocal fold lesions[14, 15]. This limitation in sampling has impeded our ability to study the role microbes play in laryngeal health and disease.

In this study, we used excised larynges from healthy pigs to compare laryngeal sampling methods and determine if there were significant differences in the mucosal microbiota between methods and sites within each individual specimen. We compared biopsy to swab, as well as vocal fold tissue to tissue immediately adjacent to the vocal fold (false vocal fold). We hypothesized that regardless of sampling method and tissue site, individual specimens would yield similar microbial populations.

## Methods

### Sample collection, DNA extraction, and PCR

**Fig 1:**
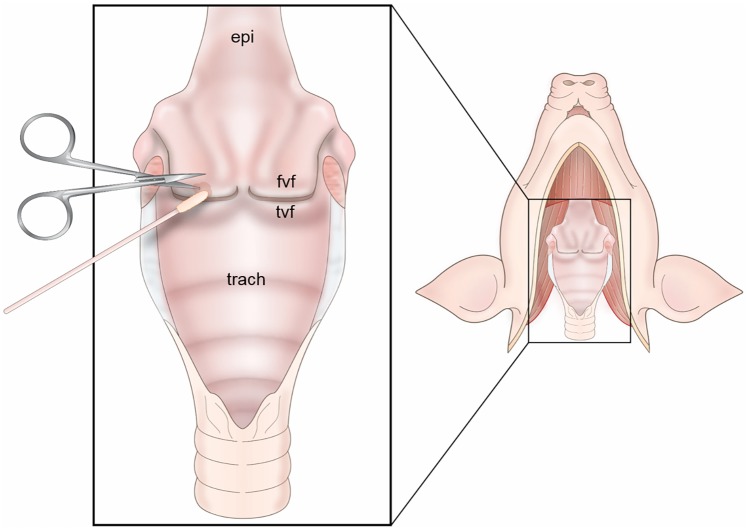
Schematic depicts the relative location of true (tvf) and false (fvf) vocal folds within the pig, with the epiglottis (epi) located closer to the mouth, and the trachea (trach) located posterior to the vocal folds. Swabs and biopsied tissue samples were taken from both true and false vocal folds on both left and right sides.

Six fresh porcine larynges were collected from two local slaughterhouses (Hoseley’s Meats, New Glarus, WI; Black Earth Meats, Black Earth, WI) in accordance with approved protocols from the Animal Care and Use Committee at the University of Wisconsin-Madison. Specimens were transported in individual bags on ice and processed by the same individual immediately upon return to the laboratory. Eight samples in total were collected from each larynx using sterile instruments, including swabs (sterile Catch-All Sample Collection Swab, EpiCentre, Madison, WI) and biopsies of the bilateral true and false vocal folds (Fig 1). Samples were immediately placed into sterile screw top tubes containing 150–200 mg of 400 μL silica beads and 300 μL of Tissue and Cell Lysis solution (Epicenter MasterPure Complete DNA and RNA extraction kit). DNA extraction and PCR were completed as found in Hanshew, Jetté, Thibeault[14]. Triplicate PCR samples were pooled together and each sample was run on a low melt agarose gel. The band of appropriate length for each sample was excised by visualization on a blue light transilluminator (Clare Chemical Research, Dolores, CO) and PCR products were extracted using the ZymoClean kit protocol (Zymo Research, Irvine, CA). The cleaned PCR products were quantified using a Qubit fluorometer (Invitrogen, Grand Island, NY). Samples were diluted and pooled at equal concentrations for 454 pyrosequencing. Two negative controls were also sequenced, including one blank DNA extraction carried through as a sample and one PCR blank with no template DNA added.

### Pyrosequencing, data processing, and statistics

454 pyrosequencing was conducted on a Roche GS Junior (Roche, Indianapolis, IN) using titanium chemistry and long read modifications found in Hanshew *et al*[16]. Samples were sequenced across four picotiter plates. Sequence data was processed as found in Hanshew, Jetté, Thibeault[14], but using the most up to date mothur v1.36.1[17], Silva (release 123; [18]), and RDP (release 14; [19)]. Initial assessment of the control samples was done by assigning sequences to operational taxonomic units (OTUs) at 97% sequence identity. These were used to construct a distance matrix using theta Yue and Clayton (theta YC) values, and calculate unweighted and weighted UniFrac[20] distances. All three were visualized with principal coordinates analysis (PCoA) plots in Prism (GraphPad, La Jolla, CA). Control samples were removed from further analysis. Using the default settings for sub.sample in mothur, communities were subsampled to 750 sequences, the lowest number of sequences in a sample, for the remainder of analyses to eliminate skew from uneven sequence depth. Chao, inverse Simpson, Shannon’s diversity, and Good’s coverage were calculated in mothur. One-way ANOVA with TukeyHSD *p* value correction for pairwise comparison was used to assess differences between swab and biopsy, true and false, left and right, and slaughterhouse origin for Chao, inverse Simpson, and Shannon. The remaining sequences for pig samples were used to construct a new theta YC distance matrix and a new PCoA plot. Metastats[21] implemented in mothur was used to determine which, if any, operational taxonomic units (OTUs) were statistically significantly different in samples at *P* < 0.05. To test if communities were significantly different, we used permutational analysis of variance (PERMANOVA; PRIMER-E v. 7; Auckland, New Zealand; 999 permutations) based on the theta YC distance matrix using true/false vocal fold, left/right, and swab/biopsy as fixed effects. Individual pigs were included as random effect. Sequences were submitted to NCBI sequence read archive, PRJNA354188.

## Results

166,334 high quality sequences resulted from four picotiter plates after data processing. One sample, Pig5swabLT (Pig 5, swab, left, true), resulted in only five sequences, and was removed from further analysis due to few resulting sequences. The initial analysis included two negative control samples, one from a DNA extraction that did not include an actual sample, and one from a PCR with no added DNA template. As expected[22], both samples resulted in bacterial sequences, though community composition was different than the total community in pig samples, whether they were compared with PCoAs constructed from theta YC values, or unweighted or weighted UniFrac (S1 Fig). Controls were removed from further analysis.

In total, 164,196 sequences were distributed across 47 successful samples with an average of 3493.5 sequences per sample (range 750–11602). The average Good’s coverage was 98.0%, with the lowest 89.6%, suggesting sufficient coverage of communities.

Values for Chao 1 richness estimates (range 48.90–355.67, mean 139.37), inverse Simpson index (range 1.48–40.00, mean 16.44), and Shannon’s diversity indices (range 0.90–4.12, mean 3.21) (Table 1) were not significantly different for samples from the two slaughterhouses, nor did comparisons differ significantly between samples taken from the left and ride sides. The mean Chao values for swabs were significantly higher than biopsies (P<0.001), but only the comparison for Pig 2 biopsy versus Pig 2 swab was significantly different (P<0.01) amongst individual pigs. Shannon’s diversity values and inverse Simpson did not differ significantly based on sampling technique. Samples taken from true and false vocal folds were not significantly different for the three diversity metrics, however the values for both Simpson and Shannon were significantly lower for Pig 3 false vocal folds versus Pig 3 true vocal folds (P<0.01).

**Table 1:** Mean of sequence diversity.

	Chao	1/Simpson	Shannon
All Biopsies	97.97^a^	14.95	3.08
All Swabs	182.56^a^	17.98	3.35
Pig1Biopsy	92.76	21.28	3.41
Pig1Swab	153.87	24.03	3.70
Pig2Biopsy	104.18^b^	6.52	2.45
Pig2Swab	234.94^b^	9.43	2.97
Pig3Biopsy	85.17	14.15	2.63
Pig3Swab	166.90	18.92	2.82
Pig4Biopsy	74.82	12.95	3.06
Pig4Swab	166.19	14.91	3.38
Pig5Biopsy	82.64	14.47	3.19
Pig5Swab	146.91	25.87	3.68
Pig6Biopsy	148.22	20.34	3.70
Pig6Swab	217.67	16.71	3.61
True Vocal Folds	136.29	16.47	3.22
False Vocal Folds	142.31	16.41	3.20
Pig1True	115.74	19.95	3.41
Pig1False	130.90	25.36	3.70
Pig2True	130.61	3.92	2.08
Pig2False	208.51	12.03	3.34
Pig3True	151.08	30.65^c^	3.85^d^
Pig3False	101.00	2.41^c^	1.60^d^
Pig4True	106.86	10.85	3.08
Pig4False	134.14	17.02	3.37
Pig5True	113.47	16.62	3.39
Pig5False	107.73	21.41	3.40
Pig6True	194.29	16.85	3.55
Pig6False	171.59	20.20	3.76
Left	133.52	16.07	3.09
Right	144.96	16.78	3.32
Pig1Left	123.72	24.83	3.64
Pig1Right	122.92	20.47	3.47
Pig2Left	169.95	9.06	2.46
Pig2Right	169.17	6.89	2.96
Pig3Left	137.76	17.51	2.64
Pig3Right	114.31	15.56	2.81
Pig4Left	123.69	12.67	3.21
Pig4Right	117.32	15.19	3.24
Pig5Left	99.89	20.42	3.31
Pig5Right	117.91	18.56	3.47
Pig6Left	137.74	13.02	3.34
Pig6Right	228.14	24.03	3.97
Black Earth	153.13	20.59	3.61
Hoseley's	132.26	14.29	3.00

Mean Chao, inverse Simpson, and Shannon are reported for the various comparisons between biopsies and swabs, true and false, left and right, and the two slaughterhouse sources.

^a, b, c, d^: Values found to be significantly different.

**Fig 2:**
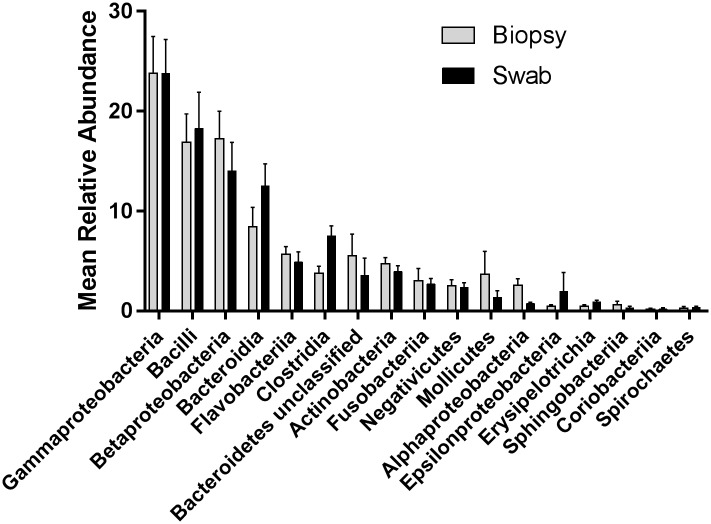
Taxonomic composition in biopsy and swab samples. Mean relative abundance in the fourteen most common classes. Error bars represent standard error.

Communities were dominated by six phyla commonly found in animals, including, in order of mean abundance, Proteobacteria (42.47%), Firmicutes (26.35%), Bacteroidetes 20.72%), Actinobacteria (4.56%), Fusobacteria (2.88%), and Tenericutes (2.55%). Fourteen classes of bacteria explained most of this diversity, including Actinobacteria, Alphaproteobacteria, Bacilli, Bacteroidia, Bacteroidetes (unclassified), Betaproteobacteria, Clostridia, Epsilonproteobacteria, Erysipelotrichia, Flavobacteriia, Fusobacteriia, Gammaproteobacteria, Mollicutes, and Negativicutes (Fig 2). While Gammaproteobacteria were the most dominant members of these communities (mean 23.79%), only *Streptococcus*, a genus in Bacilli, was present in every sample (mean 12.98%).

**Fig 3:**
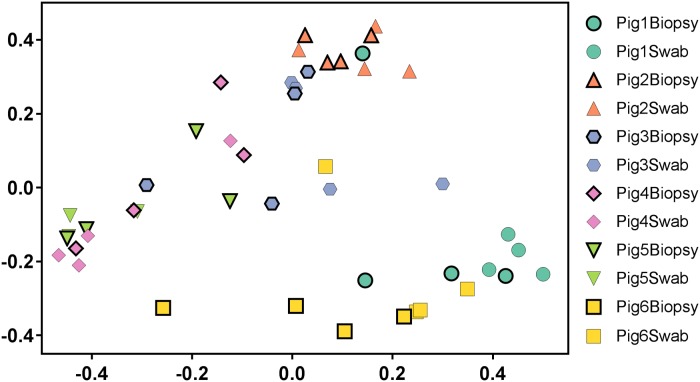
Comparison of bacterial community structure in pig biopsy and swab samples. PCoA based on theta YC distances. Biopsy samples are shown with thicker borders while swabs are shown with thinner boarders.

Communities did not cluster in the PCoA plot (Fig 3) based on sampling method (swab vs. biopsy; PERMANOVA; p = 0.77) or sampling site (true vocal fold vs. false vocal fold; PERMANOVA; p = 0.35). However, individual pigs were found to cluster together (PERMANOVA; p = 0.007). This lack of differentiation was further supported by the results of metastats. Out of the top 100 OTUs, only ten differed significantly between communities from biopsies and communities from swabs (Table 2; S1 Table). Six OTUs were present in higher average abundance in biopsy samples while four were higher in swab samples. These OTUs broadly represent four phyla (Actinobacteria, Bacteroidetes, Firmicutes, and Proteobacteria) commonly found in animals, and ten different genera (S1 Table). Similarly, some OTUs differed between biopsies and swabs in individual pigs, seven for Pig 1, five for Pig 2, five for Pig 3, twelve for Pig 4, two for Pig 5, six for Pig 6 (S1 Table).

**Table 2:** OTUs identified by metastats as significantly different between sampling methods.

Phyla/OTU#		Biopsy	Swab	*P* value
Actinobacteria				
077	Actinobacteria;Actinomycetales;Corynebacteriaceae;Corynebacterium	0.11 ± 0.10	0.03 ± 0.02	**0.004**
Bacteroidetes				
063	Flavobacteriia;Flavobacteriales;Flavobacteriaceae;Flavobacteriaceae_unclassified	0.08 ± 0.03	0.35 ± 0.15	**0.044**
Firmicutes				
029	Clostridia;Clostridiales;Clostridiaceae_1;Clostridium_sensu_stricto	0.59 ± 0.18	1.35 ± 0.30	**0.041**
067	Erysipelotrichia;Erysipelotrichales;Erysipelotrichaceae;Turicibacter	0.14 ± 0.07	0.38 ± 0.09	**0.03**
092	Negativicutes;Selenomonadales;Veillonellaceae;Megasphaera	0.04 ± 0.03	0.13 ± 0.05	**0.01**
Proteobacteria				
046	Alphaproteobacteria;Rhizobiales;Methylobacteriaceae;Methylobacterium	0.50 ± 0.27	0.01 ± 0.01	**0.001**
052	Alphaproteobacteria;Sphingomonadales;Sphingomonadaceae;Sphingobium	0.62 ± 0.19	0.08 ± 0.03	**0.001**
041	Betaproteobacteria;Burkholderiales;Burkholderiaceae;Ralstonia	0.84 ± 0.19	0.09 ± 0.03	**0.001**
069	Betaproteobacteria;Burkholderiales;Comamonadaceae;Variovorax	0.66 ± 0.44	0.05 ± 0.04	**0.048**
019	Gammaproteobacteria;Pseudomonadales;Pseudomonadaceae;Pseudomonas	2.47 ± 0.60	0.46 ± 0.14	**0.001**

Averages are from percent relative abundance. Relative abundance (%) mean ± SE.

Out of the top 100 OTUs, only five differed significantly between communities from true vocal folds and communities from false vocal folds (Table 3; S2 Table). Four OTUs were present in higher average abundance in true vocal fold samples while one was higher in false vocal fold samples. Similarly, some OTUs differed between true vocal folds and false vocal folds in individual pigs, two for Pig 1, five for Pig 2, twelve for Pig 3, one for Pig 4, four for Pig 5, and four for Pig 6.

**Table 3:** OTUs identified by metastats as significantly different between the true and false vocal folds.

Phyla/OTU#		True Vocal Folds	False Vocal Fold	*P* value
Actinobacteria				
077	Actinobacteria;Actinomycetales;Corynebacteriaceae;Corynebacterium	0.15 ± 0.11	0 ± 0	**0.028**
Bacteroidetes				
094	Flavobacteriia;Flavobacteriales;Flavobacteriaceae;Chryseobacterium	0.10 ± 0.08	0.04 ± 0.03	**0.040**
Firmicutes				
083	Negativicutes;Selenomonadales;Veillonellaceae;Veillonella	0.19 ± 0.18	0 ± 0	**0.034**
092	Negativicutes;Selenomonadales;Veillonellaceae;Megasphaera	0.05 ± 0.03	0.12 ± 0.04	**0.044**
Proteobacteria				
051	Gammaproteobacteria;Pseudomonadales;Moraxellaceae;Acinetobacter	0.49 ± 0.12	0.21 ± 0.07	**0.035**

Averages are from percent relative abundance. Relative abundance (%) mean ± SE.

## Discussion

Comparative microbial community studies of the healthy and diseased human larynx are difficult to undertake due to ethical considerations in procuring vocal fold tissue. In this study, we used a limited sample size of excised pig larynges to compare tissue biopsies and swabs, and determine if less invasive microbial sampling via swab would produce similar results to mucosal biopsy. We further examined differences in microbial communities between two laryngeal sites with distinct functions (i.e., true and false vocal folds) to explore the utility of sampling non-voice-producing tissue as a valid surrogate for the vocal folds. Results of this study provide evidence that less damaging techniques for sampling the larynx are reasonable for investigating the laryngeal microbiome, and this work should be piloted in excised human larynges to verify findings.

The communities found in these pigs were similar to those that have been described in both human false vocal fold biopsies and in lesion samples[11, 14]. Communities were comprised of phyla commonly associated with animals including Actinobacteria, Bacteroidetes, Firmicutes, Fusobacteria, Proteobacteria, and Tenericutes. Interestingly, while Gammaproteobacteria were on average the most abundant members of these communities (Fig 2), an OTU from the class Bacilli identified as *Streptococcus* was the only community member present in every sample. Other OTUs identified to the genera level were also common between these pig samples and those found in humans, such as *Cloacibacterium* and an unclassified Comamonadaceae, suggesting that maybe beyond just structural similarities between pig and human larynges, that there may be broader similarities in their microbial communities.

Overall, alpha diversity metrics did not vary significantly between our sample techniques nor locations. However, Chao 1 richness estimates were significantly different between swab and biopsy samples, despite only samples from Pig 2 swab and biopsy differing in individual pigs (Table 1). There could be a few reasons why this measure of alpha diversity differed significantly between our two tested sampling techniques. Chao 1 values are particularly sensitive to singletons and rare taxa, and more rare taxa could exist at the outer surface of the mucosal surface for the vocal folds. Though perhaps more likely is that exactly how samples were taken influenced these values. In all cases, swab samples were taken first at each site, then biopsies, which could have stripped a significant portion of the bacterial community away, leaving less behind for the biopsy sample. If more rare taxa exist in the very outer layers of the mucosa, this could explain this significant difference. Less easily explained is why only the values for Pig 3 true and false vocal folds differed for Shannon and inverse Simpson. Regardless of this, the lack of many differences for these measures of richness and evenness suggest that the less invasive sampling using swabs, or false vocal folds, is an equivalent means to sample communities in these delicate tissues.

Similarly, the measure of beta diversity was not significantly different between our sample techniques nor locations. Overall, communities from individual samples tended to cluster with other samples from the same pig (Fig 3). While minor differences could be found (Tables 2 and 3) out of the 100 most common OTUs within samples, most did not vary considerably. Also of note is that even within these differences, most of the OTUs that were found to be significantly different between sampling technique (swab versus biopsy) or location (true versus false) are relatively minor components of the community, most being present as less than 1% of the total community. This further supports that the communities found using swabs and biopsies do not differ significantly and that these two sampling techniques could be used interchangeably, much as true and false vocal folds could be used as proxies for each other when sampling.

An ideal study of shifts in laryngeal microbiota associated with disease states would include experimental controls such as biological replicates, disease-free samples, and samples with a known microbial population. To date, there are four published studies examining laryngeal microbiota using next-generation sequencing technology[11, 13–15], and most of these studies are limited by lack of a true control group. Further, given the paucity of data, there is no consensus on sampling methodology. Gong et al.[15] were the first to publish on microbial communities in the larynx by comparing the microbiota of tumor tissue collected from patients with laryngeal cancer, cancer-free laryngeal tissue adjacent to the site of the tumor from the same patients, and vocal fold polyp lesions procured from a control patient population. Though they reported differences in relative abundance of 15 genera in cancer versus “control”, their control group was not disease-free given the presence of benign disease, nor were the samples procured from the same anatomical site. In their second study, Gong et al.[13] again compared the microbiota of laryngeal cancer specimens to vocal fold polyps, sampling from two sites using two sampling methods. Their data appear to show distinct microbial communities in swabs versus biopsies; however, they collected swabs from a different location (upper throat near epiglottis) in the larynx than where they collected tissues (vocal fold), confounding interpretation of these data. In a paper examining microbial communities of benign vocal fold lesions[14], all samples were tissues removed from the vocal folds as part of the standard of care and there was no control group because of the ethical implications of removing healthy vocal fold. Jetté et al.[11] studied microbial shifts associated with smoking and reflux in otherwise healthy participants by examining tissue biopsies of the false vocal folds collected under local anesthesia. Given the results of the current study, swabbing the true or false vocal folds to sample the laryngeal microbiota would be a useful tool for comparing health and disease. Moreover, control replicates could be obtained by swabbing the vocal folds at multiple time points.

The question of adequate sampling technique for studying microbial populations that may be implicated in disease has been addressed in other body sites including sinus, esophagus, and rectum. In the sinus, it has been hypothesized that bacteria associated with chronic rhinosinusitis adhere to or exist within the sinus mucosa[23], suggesting that sampling via mucosal swab would fail to capture the extent of the microbial populations associated with disease. Exploring this possibility, Bassiouni *et al*.[7] compared the microbiota of paired mucosal tissue and swab samples collected from six patients with sinus disease and found no difference in sampling methods. Results comparing two sampling techniques used for investigating the esophageal microbiome–secretions collected from a string and mucosal biopsy–also demonstrated similarities in microbial profiles[24]. In contrast, there were significant differences in bacterial profiles of rectal swabs compared to rectal mucosal biopsies, potentially due to contamination of swabs with skin microbiota during sampling[5]. Because of our findings for laryngeal sampling, given sampling types yield similar results, it would be possible to obtain a laryngeal swab in an awake patient in the office using transnasal or transoral surgical tools. Obtaining swabs from immobilized surgical patients using direct microscopic visualization of the larynx would limit contamination from other sites such as nose, sinus, pharynx, and mouth, and possibly yield the most accurate microbial profiles.

This study was designed to identify correlations between microbial communities sampled from different anatomical sites using two sampling techniques and not to characterize the laryngeal microbiome of pigs. We have, therefore, refrained from reporting on taxa level OTU assignments, though these data are publically available and could be used for later analysis.

## Conclusion

Our results indicate that there is no difference in laryngeal microbial communities when sampled via swab or biopsy from either the true or false vocal fold in an excised pig larynx. We conclude that swabbing the vocal folds or false vocal folds would provide equivocal results to biopsy of either of these sites for future examination of the laryngeal microbiome.

## Supporting information

S1 Fig Comparison of bacterial sequences in pig biopsy and swab samples with PCoAs constructed from theta YC values, unweighted UniFrac and weighted UniFrac. Panel A, theta YC PCoA; Panel B, unweighted UniFrac PCoA; Panel C, weighted UniFrac PCoA.
(TIF)

S1 Table Significant OTUs between communities from biopsies and communities from swabs. (PDF)

S2 Table Significant OTUs between communities from true vocal folds and communities from false vocal folds. (PDF)
