# Supplementary figures and images for: A comparison of sampling methods for examining the laryngeal microbiome
Source: PLoS One. 2017 Mar 31;12(3):e0174765. doi: 10.1371/journal.pone.0174765 (PMC5375147; doi:10.1371/journal.pone.0174765)

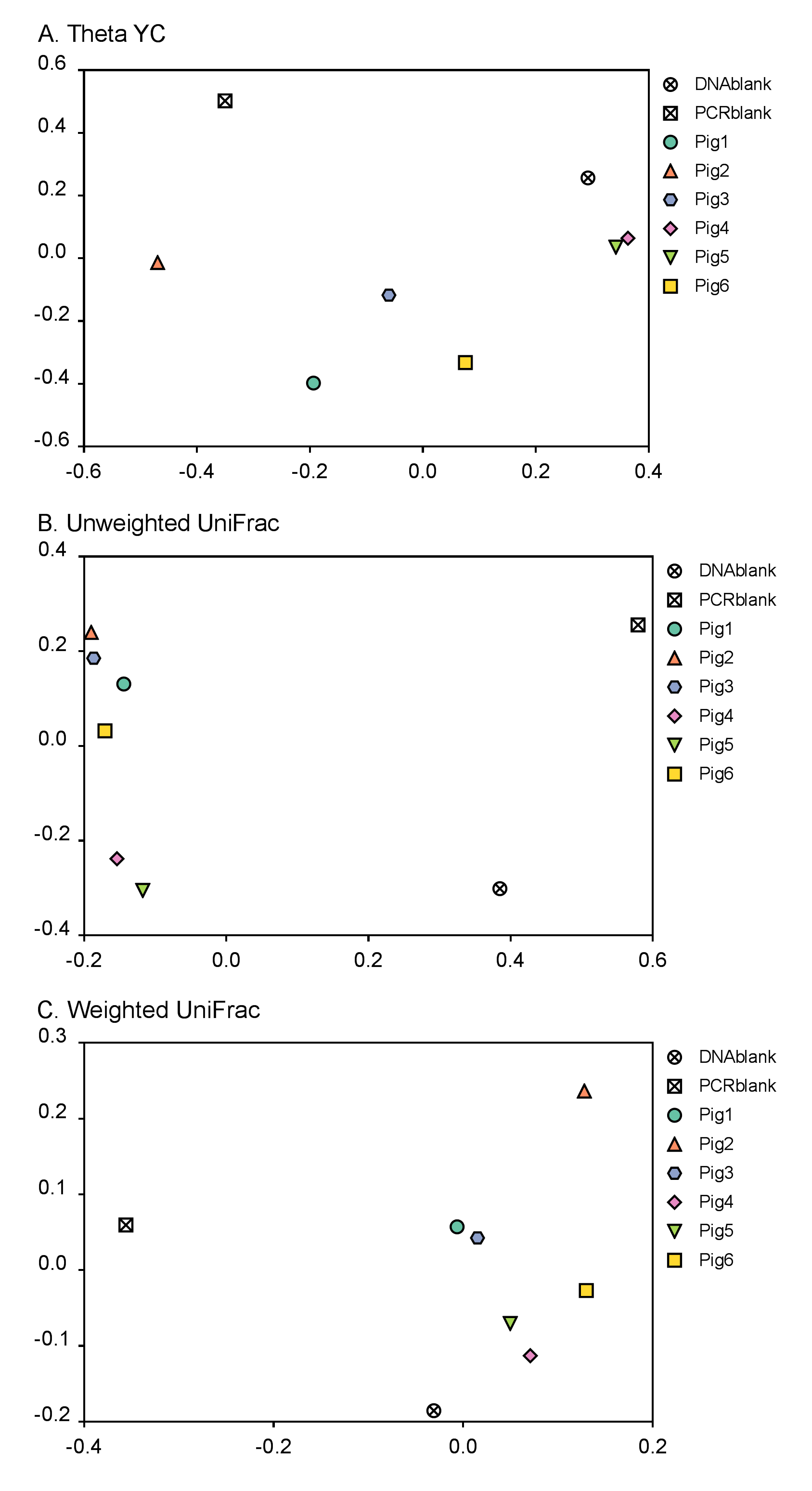

Supplement: S1 Fig — Panel A, theta YC PCoA; Panel B, unweighted UniFrac PCoA; Panel C, weighted UniFrac PCoA. (TIF) [file pone.0174765.s001.tif]
