# Supplementary material for: A comparison of sampling methods for examining the laryngeal microbiome
Source: PLoS One. 2017 Mar 31;12(3):e0174765. doi: 10.1371/journal.pone.0174765 (PMC5375147; doi:10.1371/journal.pone.0174765)
Supplement: S1 Table — (PDF) [file pone.0174765.s002.pdf]

Otu00003
Otu00006
Otu00007
Otu00008
Otu00013
Otu00014
Otu00017
Otu00019
Otu00021
Otu00024
Otu00029
Otu00030
Otu00038
Otu00040
Otu00041
Otu00046
Otu00049
Otu00051
Otu00052
Otu00055
Otu00056
Otu00060
Otu00061
Otu00063
Otu00066
Otu00067
Otu00068
Otu00069
Otu00077
Otu00079
Otu00087
Otu00088
Otu00089
Otu00092
Otu00098

Taxonomy
Bacteroidetes(100);Flavobacteriia(100);Flavobacteriales(100);Flavobacteriaceae(100);Cloacibacterium(100);
Proteobacteria(100);Betaproteobacteria(100);Burkholderiales(100);Comamonadaceae(100);Acidovorax(99);
Bacteroidetes(100);Sphingobacteriia(100);Sphingobacteriales(100);Chitinophagaceae(99);Chitinophagaceae_uncla
Proteobacteria(100);Betaproteobacteria(100);Burkholderiales(100);Comamonadaceae(100);Diaphorobacter(51);
Bacteroidetes(100);Bacteroidia(100);Bacteroidales(100);Bacteroidaceae(100);Bacteroides(100);
Proteobacteria(100);Betaproteobacteria(100);Burkholderiales(100);Comamonadaceae(100);Comamonadaceae_u
Bacteroidetes(100);Bacteroidia(99);Bacteroidales(99);Prevotellaceae(97);Prevotellaceae_unclassified(72);
Proteobacteria(100);Gammaproteobacteria(100);Pseudomonadales(100);Pseudomonadaceae(100);Pseudomonas
Bacteroidetes(100);Bacteroidia(100);Bacteroidales(100);Prevotellaceae(100);Prevotella(100);
Proteobacteria(100);Epsilonproteobacteria(100);Campylobacterales(100);Helicobacteraceae(100);Helicobacter(10
Firmicutes(100);Clostridia(100);Clostridiales(100);Clostridiaceae_1(100);Clostridium_sensu_stricto(100);
Firmicutes(100);Bacilli(100);Bacillales(100);Bacillaceae_1(91);Bacillus(91);
Firmicutes(100);Bacilli(100);Lactobacillales(100);Streptococcaceae(100);Streptococcus(100);
Proteobacteria(100);Gammaproteobacteria(100);Pasteurellales(100);Pasteurellaceae(100);Haemophilus(96);
Proteobacteria(100);Betaproteobacteria(100);Burkholderiales(100);Burkholderiaceae(100);Ralstonia(98);
Proteobacteria(100);Alphaproteobacteria(100);Rhizobiales(100);Methylobacteriaceae(100);Methylobacterium(10
Proteobacteria(100);Gammaproteobacteria(100);Pasteurellales(100);Pasteurellaceae(100);Actinobacillus(85);
Proteobacteria(100);Gammaproteobacteria(100);Pseudomonadales(100);Moraxellaceae(100);Acinetobacter(100)
Proteobacteria(100);Alphaproteobacteria(100);Sphingomonadales(100);Sphingomonadaceae(100);Sphingobium(
Bacteroidetes(100);Bacteroidia(100);Bacteroidales(100);Prevotellaceae(100);Prevotella(100);
Firmicutes(100);Bacilli(100);Lactobacillales(100);Aerococcaceae(100);Globicatella(100);
Firmicutes(100);Clostridia(100);Clostridiales(100);Peptostreptococcaceae(100);Clostridium_XI(100);
Proteobacteria(100);Betaproteobacteria(100);Burkholderiales(100);Alcaligenaceae(100);Castellaniella(100);
Bacteroidetes(100);Flavobacteriia(100);Flavobacteriales(100);Flavobacteriaceae(100);Flavobacteriaceae_unclassi
Proteobacteria(100);Gammaproteobacteria(100);Pseudomonadales(100);Pseudomonadaceae(100);Pseudomonas
Firmicutes(100);Erysipelotrichia(100);Erysipelotrichales(100);Erysipelotrichaceae(100);Turicibacter(100);
Firmicutes(100);Clostridia(100);Clostridiales(100);Peptostreptococcaceae(100);Peptostreptococcus(100);
Proteobacteria(100);Betaproteobacteria(100);Burkholderiales(100);Comamonadaceae(100);Variovorax(94);
Actinobacteria(100);Actinobacteria(100);Actinomycetales(100);Corynebacteriaceae(100);Corynebacterium(100);
Proteobacteria(100);Gammaproteobacteria(100);Enterobacteriales(100);Enterobacteriaceae(100);Enterobacteria
Actinobacteria(100);Actinobacteria(100);Actinomycetales(100);Actinomycetaceae(100);Actinomyces(100);
Firmicutes(100);Clostridia(100);Clostridiales(100);Peptostreptococcaceae(100);Clostridium_XI(100);
Firmicutes(100);Bacilli(100);Lactobacillales(100);Lactobacillaceae(94);Lactobacillus(89);
Firmicutes(100);Negativicutes(100);Selenomonadales(100);Veillonellaceae(100);Megasphaera(100);
Firmicutes(100);Bacilli(100);Lactobacillales(100);Enterococcaceae(100);Enterococcus(100);

	AllBiopsy (avg)	SE	AllSwab (avg)	SE	p-value	Pig1swab (avg)	SE	Pig1biopsy (avg)
						0.467	0.058	2.900
assified(99);								
						8.100	0.695	3.500
nclassified(99);								
	2.472	0.600	0.464	0.140	0.001			
0);						0	0	2.233
	0.594	0.178	1.345	0.295	0.041			
	0.839	0.187	0.093	0.034	0.001			
	0.500	0.270	0.006	0.006	0.001			
;								
	0.622	0.190	0.081	0.033	0.001			
						0.133	0.027	0
	0.083	0.028	0.354	0.145	0.044	0.700	0.117	0.067
s(99);						0.033	0.017	0.233
	0.139	0.066	0.377	0.088	0.030	0.800	0.139	0.100
	0.661	0.440	0.052	0.042	0.048			
	0.111	0.100	0.029	0.020	0.004			
ceae_unclassified(63);								
	0.044	0.025	0.128	0.046	0.010			

SE	p-value	Pig2swab (avg)	SE	Pig2biopsy (avg)	SE	p-value	Pig3swab (avg)	SE
0.408	0.009							
0.649	0.021							
							2.000	0.418
		0.267	0.133	1.633	0.228	0.023		
0.333	0.006							
		0.133	0.027	0.000	0.000	0.023		
		0.067	0.033	0.800	0.136	0.019	0.033	0.017
							0.267	0.067
		0	0	0.233	0.032	0.004		
							0.067	0.033
0	0.020							
0.019	0.015							
0.042	0.035							
0.050	0.024							
		0.567	0.120	0.033	0.017	0.039		
							0.700	0.100

Pig3biopsy (avg)	SE	p-value	Pig4swab (avg)	SE	Pig4biopsy (avg)	SE	p-value
			11.533	0.835	7.200	0.596	0.039
			6.367	0.450	3.567	0.326	0.018
0.533	0.105	0.048					
			0.633	0.107	1.433	0.145	0.033
			0.600	0.145	0	0	0.042
			2.433	0.212	1.333	0.141	0.038
			0.133	0.027	0	0	0.018
			0.600	0.019	1.567	0.160	0.007
0.933	0.233	0.030					
0	0	0.026					
0.767	0.191	0.038					
			0.700	0.074	0.033	0.017	0.001
			1.133	0.079	0.267	0.027	0.001
			0.467	0.079	0.067	0.019	0.020
			0.200	0.043	0	0	0.026
0.067	0.033	0.003					
			0.367	0.083	0	0	0.034

Pig5swab (avg)	SE	Pig5biopsy (avg)	SE	p-value	Pig6swab (avg)	SE	Pig6biopsy (avg)
					2.200	0.306	6.633
					3.200	0.450	10.767
					0.567	0.110	0.067
					0.367	0.032	0.800
0	0	0.400	0.113	0.044			
					0.033	0.017	0.400
					0.567	0.050	0.133
0	0	0.833	0.179	0.016			

SE	p-value
0.846	0.021
1.529	0.025
0.033	0.043
0.072	0.016
0.047	0.004
0.047	0.010
