# Supplementary material for: A comparison of sampling methods for examining the laryngeal microbiome
Source: PLoS One. 2017 Mar 31;12(3):e0174765. doi: 10.1371/journal.pone.0174765 (PMC5375147; doi:10.1371/journal.pone.0174765)
Supplement: S2 Table — (PDF) [file pone.0174765.s003.pdf]

	Taxonomy	AllTrue (avg)	SE	AllFalse (avg)	SE	p-value
Otu00001	Firmicutes(100);Bacilli(100);Lactobacillales(100);Streptococcaceae(100);Strepto					
Otu00002	Proteobacteria(100);Gammaproteobacteria(100);Pseudomonadales(100);Mora					
Otu00003	Bacteroidetes(100);Flavobacteriia(100);Flavobacteriales(100);Flavobacteriaceae					
Otu00005	Proteobacteria(100);Gammaproteobacteria(100);Pseudomonadales(100);Mora					
Otu00007	Bacteroidetes(100);Sphingobacteriia(100);Sphingobacteriales(100);Chitinophag					
Otu00008	Proteobacteria(100);Betaproteobacteria(100);Burkholderiales(100);Comamona					
Otu00009	Proteobacteria(100);Gammaproteobacteria(100);Pasteurellales(100);Pasteurell					
Otu00014	Proteobacteria(100);Betaproteobacteria(100);Burkholderiales(100);Comamona					
Otu00016	Proteobacteria(100);Betaproteobacteria(100);Burkholderiales(100);Burkholderi					
Otu00018	Actinobacteria(100);Actinobacteria(100);Actinomycetales(100);Propionibacteria					
Otu00029	Firmicutes(100);Clostridia(100);Clostridiales(100);Clostridiaceae_1(100);Clostrid					
Otu00034	Bacteroidetes(100);Bacteroidia(100);Bacteroidales(100);Prevotellaceae(95);Allo					
Otu00039	Proteobacteria(100);Betaproteobacteria(100);Rhodocyclales(100);Rhodocyclace					
Otu00041	Proteobacteria(100);Betaproteobacteria(100);Burkholderiales(100);Burkholderi					
Otu00042	Firmicutes(100);Bacilli(100);Bacillales(100);Bacillales_Incertae_Sedis_XI(100);Ge					
Otu00045	Proteobacteria(100);Betaproteobacteria(100);Burkholderiales(100);Comamona					
Otu00048	Firmicutes(100);Bacilli(100);Lactobacillales(100);Streptococcaceae(100);Strepto					
Otu00051	Proteobacter	0.493	0.122	0.206	0.065	0.035
Otu00070	Proteobacteria(100);Gammaproteobacteria(100);Enterobacteriales(100);Entero					
Otu00072	Proteobacteria(100);Gammaproteobacteria(100);Pseudomonadales(100);Pseud					
Otu00077	Actinobacter	0.145	0.105	0	0	0.028
Otu00083	Firmicutes(1	0.186	0.180	0	0	0.034
Otu00088	Firmicutes(100);Clostridia(100);Clostridiales(100);Peptostreptococcaceae(100);					
Otu00092	Firmicutes(1	0.052	0.030	0.117	0.042	0.044
Otu00094	Bacteroidete	0.099	0.078	0.039	0.025	0.040
Otu00096	Actinobacteria(100);Actinobacteria(100);Actinomycetales(100);Corynebacteriac					

	Pig1True (av	SE	Pig1False (av	SE	p-value	Pig2True (av	SE
coccus(100);							
xellaceae(100);Moraxella(100);							
e(100);Cloacibacterium(100);							
xellaceae(100);Moraxellaceae_unclassified(100);							
aceae(99);Chitinophagaceae_unclassified(99);							
daceae(100);Diaphorobacter(51);						0.033	0.033
	0.700	0.319	3.600	1.143	0.020		
daceae(100);Comamonadaceae_unclassified(99);							
ales_incertae_sedis(100);Aquabacterium(100);						0	0
aceae(100);Propionibacterium(100);						0.167	0.084
lium_sensu_stricto(100);							
prevotella(93);							
eae(100);Dechloromonas(99);							
aceae(100);Ralstonia(98);							
	0.567	0.206	1.233	0.227	0.036		
daceae(100);Delftia(100);							
coccus(100);						0.300	0.084
bacteriaceae(100);Escherichia/Shigella(94);						0	0
lomonadaceae(100);Pseudomonas(99);							
Clostridium_XI(100);							
eae(100);Corynebacterium(100);							

Pig2False (av	SE	p-value	Pig3True (avg	SE	Pig3False (av	SE
			5.133	1.374	54.467	9.475
			4.133	2.206	0.167	0.084
			0.533	0.224	24.133	11.291
			2.367	1.176	0	0
0.300	0.114	0.032				
			0.867	0.320	0	0
0.300	0.100	0.008	1.033	0.440	0	0
1.900	0.520	0.006	3.467	1.437	0.200	0.086
			0.200	0.116	0	0
			0.900	0.485	0.067	0.039
			1.600	0.906	0	0
0.933	0.196	0.012				
			0.933	0.322	0.067	0.039
0.200	0.086	0.027				
			0.633	0.305	0	0

p-value	Pig4True (avg	SE	Pig4False (av	SE	p-value	Pig5True (avg
0.000						
						0.711
0.036						
0.022						
0.025						
0.006						
0.013						
0.016						0.578
	0.133	0.133	1.733	0.758	0.038	
0.040						1.244
0.049						
0.039						
0.006						
						0
0.022						

SE	Pig5False (av	SE	p-value	Pig6True (avg	SE	Pig6False (av
				4.133	0.514	6.967
0.194	0	0	0.007			
0.118	1.500	0.300	0.013			
				0.133	0.054	0
0.194	0.633	0.220	0.042			
0	0.233	0.033	0.001			
				0	0	0.233
				0.633	0.252	0.100

SE	p-value
1.249	0.045
0	0.022
0.100	0.027
0.100	0.054
